# Acquisition of Adult-Like TLR4 and TLR9 Responses during the First Year of Life

**DOI:** 10.1371/journal.pone.0010407

**Published:** 2010-04-28

**Authors:** Muriel Nguyen, Elke Leuridan, Tong Zhang, Dominique De Wit, Fabienne Willems, Pierre Van Damme, Michel Goldman, Stanislas Goriely

**Affiliations:** 1 Institute for Medical Immunology, Université Libre de Bruxelles, Gosselies, Belgium; 2 Centre for the Evaluation of Vaccination, Vaccine and Infectious Diseases Institute, University of Antwerp, Wilrijk, Belgium; Ludwig-Maximilians-Universität München, Germany

## Abstract

**Background:**

Characteristics of the human neonatal immune system are thought to be responsible for heightened susceptibility to infectious pathogens and poor responses to vaccine antigens. Using cord blood as a source of immune cells, many reports indicate that the response of neonatal monocytes and dendritic cells (DC) to Toll-like receptor (TLR) agonists differs significantly from that of adult cells. Herein, we analyzed the evolution of these responses within the first year of life.

**Methodology/Principal Findings:**

Blood samples from children (0, 3, 6, 9, 12 month old) and healthy adults were stimulated ex vivo with bacterial lipopolysaccharide (LPS, TLR4 agonist) or CpG oligonucleotides (TLR9 agonist). We determined phenotypic maturation of monocytes, myeloid (m) and plasmacytoid (p) DC and production of cytokines in the culture supernatants. We observed that surface expression of CD80 and HLA-DR reaches adult levels within the first 3 months of life for mDCs and 6–9 months of life for monocytes and pDCs. In response to LPS, production of TNF-α, IP-10 and IL-12p70 reached adult levels between 6–9 months of life. In response to CpG stimulation, production of type I IFN-dependent chemokines (IP-10 and CXCL9) gradually increased with age but was still limited in 1-year old infants as compared to adult controls. Finally, cord blood samples stimulated with CpG ODN produced large amounts of IL-6, IL-8, IL-1β and IL-10, a situation that was not observed for 3 month-old infants.

**Conclusions:**

The first year of life represents a critical period during which adult-like levels of TLR responses are reached for most but not all cytokine responses.

## Introduction

The characteristics of immune responses in early life are often held responsible for heightened sensitivity towards infectious agents and suboptimal responses to vaccination [1], [2]. Neonatal CD4^+^ T cells are indeed unable to mount efficient Th1-type responses to most stimuli with the exception of BCG vaccine [3], [4]. As recently reviewed [5], multiple reports have explored the function of innate immune cells at birth. The capacity of neonatal monocytes and dendritic cells (DC) to produce cytokines in response to Toll-like receptor (TLR) agonists differs significantly from that of adult cells. Several reports have noted that production of TNF-α is impaired in early life. This defect is observed only in certain experimental conditions. It was initially described in cord blood from preterm infants [6]. More recently, decreased TNF-α/IL-6 ratio at birth in response to specific TLR ligands was linked to high adenosine levels in cord blood plasma [7]. It has also long been noted that production of IL-10 is elevated in LPS-stimulated cord blood in comparison to adult samples, which can also down-modulate the production of other cytokines [8], [9].

In terms of signaling pathways, neonatal cells were shown to respond in a qualitatively different manner. TLR4 is the critical receptor of LPS and is expressed on myeloid cells. TLR4 is coupled to adaptor proteins that lead to distinct signaling pathways. The “myeloid differentiation factor 88 (MyD88)-dependent” pathway is generally similar to adults in neonatal cells, although lower MyD88 expression has also been reported in cord blood monocytes and neutrophils [10], [11]. In contrast, the “MyD88-independent” pathway involving TIR-containing adaptor inducing interferon IFNβ (TRIF) and the transcription factor interferon regulatory factor (IRF)-3 [12] was shown to be less active in early life. Indeed, impaired interaction of IRF-3 with the coactivator CREB binding protein (CBP) in neonatal blood cells exposed to LPS was associated with impaired expression of IFNβ, IFN-inducible genes (such as CXCL10) and bioactive IL-12p70 [13].

Plasmacytoid DCs (pDCs) represent a major source of type I IFNs especially in the course of viral infections or exposure to TLR7 or TLR9 ligands. Although pDCs are present in significant numbers in human cord blood, there is evidence that they produce less IFN-α upon exposure to unmethylated CpG-rich oligonucleotides (CpGs) [14]. At the molecular level, this observation was linked to impaired nuclear translocation of IRF-7 [15].

Due to ethical and technical limitations, essentially all these observations were done using cord blood as a source of neonatal immune cells. Very few studies analyzed the evolution of innate immune cells function in the first months of life. This has major implication in terms of vaccination strategy as TLR4 agonists are now used in newly-developed vaccines targeting this age group. Reports indicate that the capacity of mononuclear cells to produce adult-like levels of cytokines in response to LPS/IFN-γ is reached only later in life [8], [16].

Herein, we assessed different parameters of the response of monocytes and dendritic cell subsets to LPS and CpG during the first year of life. We used a “whole-blood” assay in order to take into account the possible implication of plasma factors on TLR responses. We observed a stepwise development of the response to TLR4 and TLR9 stimulation during this period.

## Materials and Methods

### Subjects

A prospective multi-centre study was conducted in the Province of Antwerp, Belgium, in accordance with the Helsinki Declaration and procedures established by Belgian law. The study was approved by the local ethic committees (University Hospital Antwerp, Wilrijk, St Vincentius ziekenhuis vzw, Antwerp, St Augustinus ziekenhuis, Wilrijk and ZNA middelheim ziekenhuis, Antwerp). The main goal was the description of the duration of maternal antibodies against measles in two groups: children born from vaccinated women or from women with naturally acquired immunity (Leuridan E, Hens N, Hutse V, Ieven M., Aerts M, Van Damme P. How long are neonates protected by maternal antibodies? ESPID 2009, Brussels, abstract n° 728). Written informed consent was obtained from all participants and from both parents for the participating infants. Healthy pregnant women and their healthy offspring were included starting April 2006 and follow-up lasted until November 2008. Exclusion criteria were impaired immunology in mother or child, administration of immunoglobulins or blood products during the study period, preterm delivery (<36 weeks) and low birth weight (<2400 g). A questionnaire was completed on demographics, validated vaccination history and medical history. Growth parameters, breastfeeding, day-care attendance, immunization data, episodes of illness and medication used were registered for the participants at each visit, as well as medical histories for all household members. Additionally to the phlebotomy planned for the maternal antibody study, extra venous whole blood was collected from cord blood (10 ml, n = 13) and in some infants (0.5–2 ml) at 3 months (84–99 days, n = 20), 6 months (175–189 days, n = 8), 9 months (267–282 days, n = 9) and 12 months (358–372 days, n = 14). Healthy adults and mothers (3 months post-partum) were also included (n = 10 and n = 20, respectively). All samples, except for cord blood and adult controls, were collected during home visits. All women and children were Caucasian. Parameters at birth were within the normal ranges for gestational age, weight and length at birth. as expected due to the exclusion criteria (see table 1). The weight and length were followed though the complete study as well as type of feeding. Almost all children received breastfeeding with a mean duration of 15 weeks. Due to limited amount of blood, all parameters were not measured in all samples. Hence, for some parameters, values obtained from different age groups were pooled as indicated in the legends of the figures.

**Table 1:** General characteristics of the participants.

	Mothers
Number	30
Mean age in years (SD)	29 (4)
Vaccinated against measles	13
Naturally immune to measles	17

	Children
Number	30
female/male	18/12
Mean gestational age (95% CI)	39 weeks (±0,012)
Mean birth weight in grams (SD)	3182 (466) g
Mean length at birth in cm (SD)	50(±1)
Breastfeeding (number)	26
Breastfeeding (mean duration)	15 weeks
C-section	6
Caucasian race	30

### Blood stimulation

All samples were stimulated within 5 h after blood collection. Whole blood (200 µl) was incubated at 37°C with PBS (8 µl), LPS (20 ng/ml, Sigma-Aldrich, Bornem, Belgium. We assessed the specificity for TLR4 by gene reporter assay in 293 cells stably transfected with TLR2 and TLR4) or a combination of CpG ODN 2006 and 2216 (25 µg/ml each, synthesized by Tib Molbiol, Berlin, Germany). After overnight incubation (16–18 h), culture supernatant was collected by centrifugation and stored at −20°C for cytokine determination. Blood cells were resuspended in PBS for further FACS analysis.

### FACS staining

To analyze the phenotype of circulating DC, whole blood cells were first pre-incubated with FcR-blocking reagent (6 µl/200 µl, Miltenyi Biotec, Bergisch Gladbach, Germany) for 10 min and then for 30 min at 4°C with of a cocktail of FITC-conjugated mAb specific for CD3, CD19, CD20 and CD56 (2 µl each), 3 µl of allophycocyanin (APC)-conjugated anti-CD11c mAb, 2.5 µl PeCy5-conjugated CD123, 2.5 µl of Pacific blue-conjugated CD14 and 3 µl PE-conjugated anti-CD80 (all from Becton Dickinson, Mountain View, CA) and 3 µl of PE/Texas Red-conjugated anti-HLA-DR mAb (Immunotech, Marseilles, France). After this first incubation, blood cells were incubated for 10 min at room temperature in the dark with 3 ml of FACS Lysis 1X (Becton Dickinson). Cells were then centrifuged at 1500 rpm for 10 min and resuspended in 200 µl Cytofix buffer (Becton Dickinson). Samples were then analyzed using an LX-9 cytometer (Dako Cytomation). Compensation beads (CompBeads, BD biosciences) were used for each experiment to standardize voltage settings and to generate a compensation matrix.

### Cytokine determination

IL-1β, IL-6, IL-8 TNF-α, IFN-γ, IL-12p70, IP-10, IL-10 and CXCL9 (MIG) levels in samples were diluted 1/4 (and 1/10 or 1/20 to fall within the range of the standard curve) and assessed in duplicate by multiplex bead array from Bio-Rad (Bio-Plex Pro human cytokine, Bio-Rad Laboratories, Hercules, CA) according to manufacturer's instructions. Assays were read on the Bio-Plex 200 system (Bio-Rad).

### Statistical analysis

All data were analyzed using the Graphpad Prism software. Cytokine and flow cytometry data were compared using one-way ANOVA (using the non parametric Kruskal-Wallis test) with Dunns post test for multiple comparison using adult values as a reference. All *p* values are two-sided and were considered significant when *p*<0.05.

## Results

### Surface expression of CD80 and HLA-DR molecules reaches adult levels within the first 3 months of life for monocytes and mDCs and 6–9 months of life for pDCs

**Figure 1:**
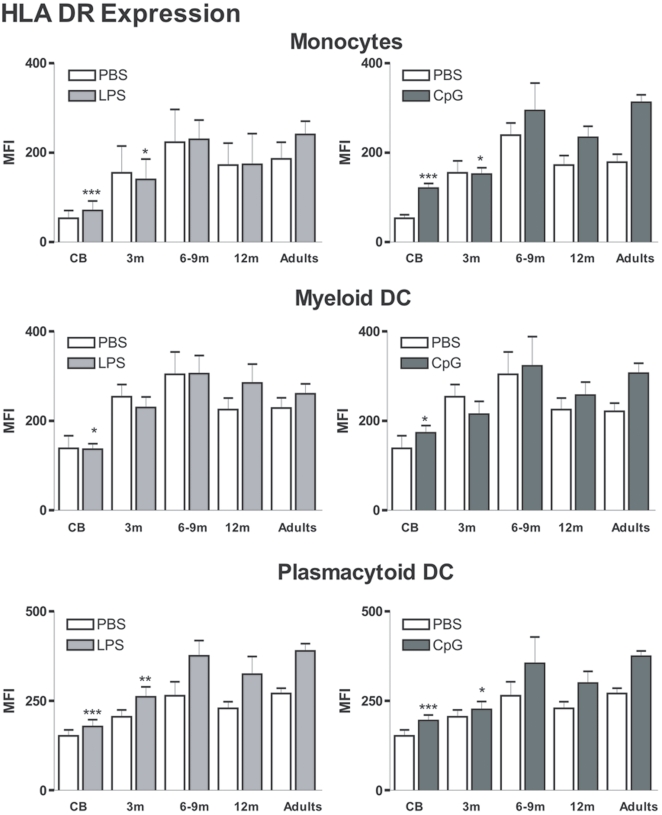
HLA DR expression on the surface of circulating monocytes, myeloid and plasmacytoid DCs. Blood samples were incubated with PBS or the indicated stimulant. The different subpopulations were gated as described in the Material and Methods section. Expression was compared in samples from the different age groups: Cord blood (CB, n = 10), 3-month (3 m, n = 10), 6&9-month (6–9 m pooled data from n = 4 and 8, respectively), 12-month (12 m, n = 9) old infants and healthy adults (n = 16). Data are represented as median+interquartile range. *p<0.05, **p<0.01, ***p<0.001 as compared to stimulated adult samples.

We first evaluated the phenotype of circulating APCs in samples obtained at birth, within the first year of life and from adult controls. Using 6-color flow cytometry, we analyzed the expression of CD80 and HLA DR molecules at the surface of monocytes (Lin-, CD14+, CD11c+), mDCs (Lin-, CD14-, CD11c+, CD123-) and pDCs (Lin-, CD14-, CD11c-, CD123+). We first noted that in cord blood, basal expression of HLA-DR molecules on monocytes, mDCs and pDCs was lower than in adult samples (Fig. 1). Within the first 6 months of life, basal HLA-DR expression gradually increased at the surface of these three populations. In LPS and CpG-stimulated adult samples, we observed a further increase in HLA DR expression at the surface of monocytes, mDCs and pDCs. For mDCs, adult-level HLA-DR expression was already reached in 3-month old infants. In contrast, for both monocytes and pDCs, HLA DR levels were significantly decreased in stimulated samples from 3-month old infants as compared to the adult group. HLA DR upregulation upon TLR4 or TLR9 stimulation in older infants (>6months) was comparable to adults.

**Figure 2:**
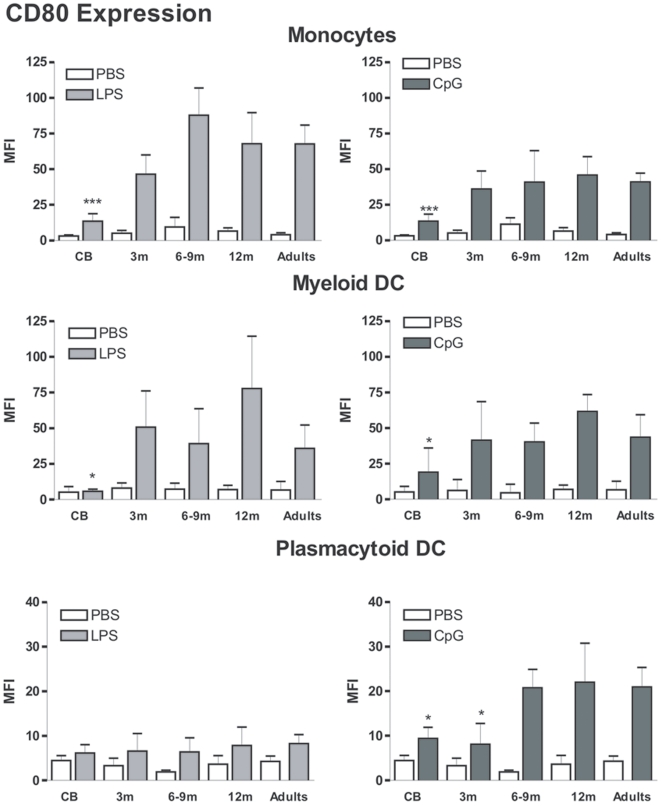
CD80 expression on the surface of circulating monocytes, myeloid and plasmacytoid DCs. The different subpopulations were gated as described in the Material and Methods section. Expression was compared in samples from the different age groups: Cord blood (CB, n = 10), 3-month (3 m, n = 10), 6 and 9-month (6–9 m, pooled data from n = 4 and 8, respectively), 12-month (12 m, n = 9) old infants and healthy adults (n = 16). Data are represented as median+interquartile range. *p<0.05, **p<0.01, ***p<0.001.

Basal CD80 expression was low in all age groups (Fig. 2). We observed marked upregulation of CD80 surface expression on monocytes and mDCs from LPS and CpG-treated adult samples. For pDCs, CpG but not LPS treatment led to an increase of CD80 expression in adult samples. This phenotypic maturation was strongly reduced in cord blood samples. For both monocytes and mDCs, LPS- and CpG-mediated upregulation of CD80 expression was comparable to adult cells for 3-, 6-, 9- and 12-months old infants. In contrast, CpG-mediated phenotypic maturation of pDCs did not reach adult-levels before the age of 6 months. These results indicate that circulating APCs acquire adult-like phenotype during the first 6 months of life. These data also suggest that this maturation process is more rapid for mDCs and monocytes than for pDCs.

### Age-dependent upregulation of LPS-induced TNF-α IP-10, IL-12p70 and IFN-γ production

**Figure 3:**
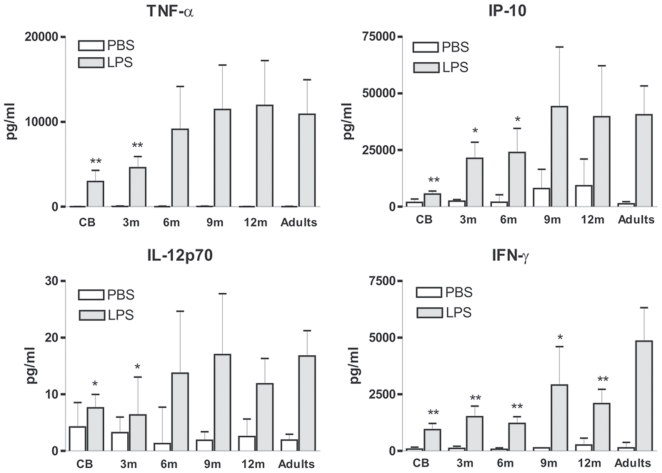
Age-dependent upregulation of LPS-induced production of TNF-α, IP-10, IL-12p70 and IFN-γ. Whole blood samples were stimulated with LPS and culture supernatants were collected after 16–18 h. Production in the different infant groups was compared to adult values. Cord blood (CB, n = 13), 3-month (3 m, n = 15), 6-month (6 m, n = 8), 9-month (9 m, n = 9), 12-month (12 m, n = 9) old infants and healthy adults (n = 10). Data are represented as median+interquartile range. *p<0.05, **p<0.01, ***p<0.001 as compared to stimulated adult samples.

We next analyzed LPS-stimulated cytokine production in whole blood. As previously described [6], [8], [9], [13], [17]–[19], we noted that cord blood cells produced reduced amounts of TNF-α, IP-10, IL-12p70 and IFN-γas compared to adult controls (Fig. 3). Adult-levels were reached at 6 months of age for TNF-α and IL-12p70. For IP-10, production significantly increased in samples collected from 3- and 6-month infants but only reaches adult levels at 9 months of age. Interestingly, IFN-γ production in response to LPS was still reduced at 1 year of age as compared to adult samples. These results indicate that the reduced capacity of cord blood cells to produce these inflammatory cytokines is not restricted to the neonatal period. However, under these experimental conditions, adult levels were reached rapidly during infancy.

### The capacity to produce type I IFN-dependent chemokines in response to CpGs is acquired progressively during the first year of life

**Figure 4:**
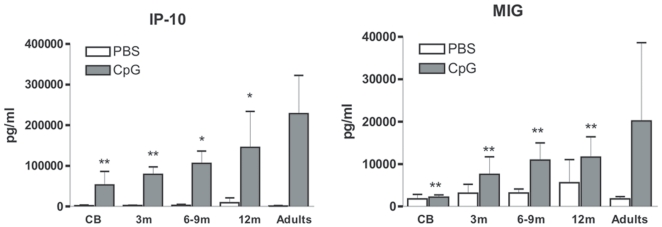
Age-dependent upregulation of CpG-induced production of IP-10 and MIG. Whole blood samples were stimulated with CpG A+B combination and culture supernatants were collected after 16–18 h. Production in the different infant groups was compared to adult values. Cord blood (CB, n = 13), 3-month (3 m, n = 10), 6&9-month (6 m, n = 3, 9 m, n = 5), 12-month (12 m, n = 11) old infants and healthy adults (n = 10). Data are represented as median+interquartile range. *p<0.05, **p<0.01, ***p<0.001 as compared to stimulated adult samples.

We next evaluated the production of cytokines induced by TLR9 agonists. We noted that production of two type I IFN-inducible chemokines, IP-10 and MIG, was strongly reduced in cord blood as compared to adult samples (Fig. 4). This is consistent with the fact that cord blood pDCs display a major defect in their capacity to produce type I IFNs [20]. We observed an age-dependent increase in the production of IP-10 and MIG but levels at 1 year of age were still significantly lower than that of adult controls. Taken together with phenotypic markers, this result suggests that maturation of pDCs function is delayed compared to that of LPS-responsive cells such as monocytes and mDCs.

### Hyperproduction of IL-6, IL-8 and IL-10 in the first months of life

**Figure 5:**
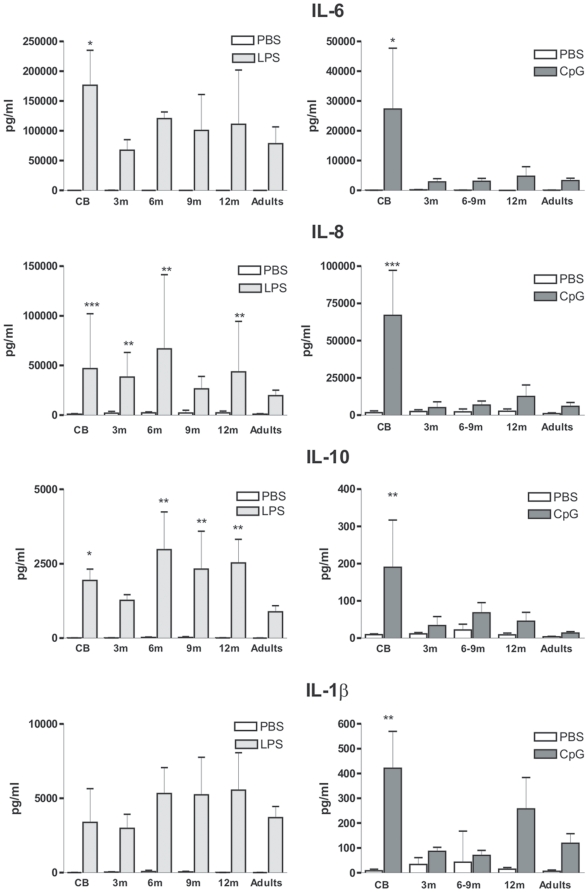
Hyperproduction of specific cytokines in early life. Whole blood samples were stimulated with the indicated TLR ligand and culture supernatants were collected after 16–18 h. Production in the different infant groups was compared to adult values. Cord blood (CB, n = 13), 3-month (3 m, n = 10), 6&9-month (6 m, n = 3, 9 m, n = 5), 12-month (12 m, n = 11) old infants and healthy adults (n = 10). Data are represented as median+interquartile range. *p<0.05, **p<0.01, ***p<0.001 as compared to stimulated adult samples.

In line with previous reports [7], [8], [19], we observed that production of several cytokines, including IL-6, IL-8 and IL-10 was significantly increased in LPS-stimulated cord blood as compared to adult samples (Fig. 5). For IL-6, production decreased to adult levels in the group of 3-month old infants and remained low in the other groups. For IL-10, production was still significantly higher in 6-, 9- and 12-month old infants as compared to adults. A similar persisting trend was also observed for IL-8 but variations between individuals were very important in these groups. Finally, no difference between the age groups was noted for IL-1β production.

Activation of adult blood through TLR9 induces low levels of inflammatory cytokines such as IL-6 and IL-8. Unexpectedly, very high levels of these cytokines were detected in CpGs-stimulated cord blood samples. This was accompanied by increased production of IL-1β and IL-10. Hyper-responsiveness to CpGs was found to be transient as it was not observed in samples collected from 3-month or older infants.

## Discussion

Over the last few years evidence has accumulated indicating that innate immune responses of newborns and adults differ significantly. Several mechanisms may contribute to these observations. It is therefore difficult to identify the developmental processes that will lead to the acquisition of adult-like responses. Herein, we focused on specific aspects of neonatal TLR responses that have previously been analyzed using cord blood. As summarized in table 2, we analyzed the ontogeny of these parameters over the first year of life. A consistent finding is the decreased capacity of cord blood-derived cells to produce bioactive IL-12p70 by monocytes and mDCs. In response to LPS, we detected low but reproducible IL-12p70 production in adult samples. By the age of 6 months, adult levels of IL-12p70 were observed in some LPS-stimulated samples. Our data is in line with a recent report that indicates that LPS+IFNγ-induced IL-12p70 production in whole blood was significantly increased between birth and 1 month of age but still low compared to adult samples [21]. A previous report indicated that decreased capacity to produce IL-12p70 was observed throughout childhood (samples from 5- and 12-yr-old children were analyzed) [16]. There are major differences in the design of the experiments that could account for this apparent discrepancy. In their work, Upham *et al* analyzed IL-12p70 production in isolated PBMC, cultured in FCS-containing medium and stimulated by LPS and IFNγ combination. Here, we analyzed IL-12p70 production in LPS-stimulated whole blood. Multiple parameters that differ between these experiments could affect the capacity of APCs to produce IL-12p70. For example, circulating plasma factors and the presence of red blood cells affect cytokine production [5], [22]. The cellular source of IL-12 could also differ. Indeed, when monocytes are primed with IFN-γ, they gain the ability to produce IL-12p70 through upregulation of IRF1 and IRF8 expression [23], [24]. Taken together, these data indicate that the capacity of circulating APCs to produce IL-12p70 in response to LPS gradually increases within the first months of life. However, this might not reflect the capacity of cells to produce IL-12 under more intense stimulation conditions. Reduced production of IFN-γ in response to LPS was observed throughout the first year of life. This is consistent with the fact that it reflects both the capacity of TLR4-expressing cells to produce IL-12 and of lymphocytes and NK cells to produce IFN-γ. Indeed, upon direct stimulation of lymphocytes by phytohemagglutinin, production of IFN-γ was found to be low in children until the age of 18 months [25].

**Table 2:** Summary of the main results.

Parameter	Stimulation	Response in CB	Reaches Adult level at:
CD80/DR expression (Monocytes)	LPS	▾	3–6 months
CD80/DR expression (mDC)	LPS	▾	3 months
CD80/DR expression (pDC)	CpG	▾	6–9 months
TNF-α	LPS	▾	6 months
IP-10	LPS	▾	9–12 months
IL-12p70	LPS	▾	6–9 months
IFN-γ	LPS	▾	>12 months
IL-10	LPS	▴	>12 months
IL-6	LPS	▴	3 months
MIG/IP-10	CpG	▾	>12 months
IL-8/IL-6/IL-1/IL-10	CpG	▴	3 months

**▾** decreased.

**▴** increased.

High circulating adenosine levels contribute to the low capacity of cord blood cells to produce TNF-α in response to LPS [7]. We observed that by the age of 6 months, TNFα production reached adult levels, suggesting that alteration in plasma factors last for more than 3 months after birth. Differences in plasma factors also contribute to low IP-10 production in LPS-stimulated cord blood. However, we showed that cell intrinsic factors are also important. Indeed, we previously showed that incomplete IRF3 activation in cord blood cells led to decreased IFNβ production and induction of IFN-dependent genes such as IP-10 [13]. IP-10 production reached adult levels by the age of 9 months. This result suggests that the differences in signalling pathways are gradually overcome before that age.

We previously reported that neonatal pDCs produce less type I IFNs [20]. As a consequence, IFN-inducible genes, such as IP-10 and MIG levels are strongly reduced upon TLR9 stimulation of cord blood. With age, we observe a gradual increase in production of these 2 chemokines. However, in 1-year old infants, levels were still significantly lower than in adults. This result strongly suggests that pDC reach adult-like function later in life than monocytes or mDCs. We observed a similar trend for phenotypic markers (Fig. 1).

As previously reported [8], [19], [26], we observed high production of IL-6, a pleiotropic cytokine, in cord blood samples as compared to adult samples. LPS-induced IL-6 production was comparable to adult levels by the age of 3 months. In contrast, we detected high IL-8 and IL-10 production in some samples from 3- to 12-month old infants. We observed a dramatic increase in IL-6, IL-8, IL-10 and IL-1β production in CpG-stimulated cord blood but not samples from older infants or adults. Interestingly, production of these cytokines by purified cord blood pDCs was not found to be increased [15]. This result suggests that an alternative cellular source of these cytokines could be more responsive to TLR9 stimulation (in a direct or indirect fashion) in cord blood. Cytokine production was found to be affected by the mode of delivery [27], [28]. However, no differences in CpG-induced cytokine levels were observed between children that were delivered naturally or through C-section. This transient overexpression of these inflammatory cytokines should be kept in mind in the context of vaccination of young infants with TLR ligand-containing adjuvants, such as monophosphoryl lipid A (MPL).

In conclusion, our study indicates that most of the parameters of TLR responses we analyzed progressively reach adult-like levels within the first year of life. Clearly, intra-uterine environment conditions the function of APCs. It remains unknown whether “maturation” of these responses reflects the normal turn-over of APCs and the disappearance of these immunomodulators or if it is an active process that requires education of immune cells by environmental exposure to microbial compounds. It would therefore be helpful to assess the impact of specific factors (vaccination, intercurrent infections, atopic background etc.) and settings (resource-rich vs. developing countries) on the ontogeny of TLR responses in early life.
